# Adult Neurogenesis Transiently Generates Oxidative Stress

**DOI:** 10.1371/journal.pone.0035264

**Published:** 2012-04-30

**Authors:** Noah M. Walton, Rick Shin, Katsunori Tajinda, Carrie L. Heusner, Jeffrey H. Kogan, Shinichi Miyake, Qian Chen, Kouichi Tamura, Mitsuyuki Matsumoto

**Affiliations:** CNS, Astellas Research Institute of America LLC, Skokie, Illinois, United States of America; University of Florida, United States of America

## Abstract

An increasing body of evidence suggests that alterations in neurogenesis and oxidative stress are associated with a wide variety of CNS diseases, including Alzheimer’s disease, schizophrenia and Parkinson’s disease, as well as routine loss of function accompanying aging. Interestingly, the association between neurogenesis and the production of reactive oxidative species (ROS) remains largely unexamined. The adult CNS harbors two regions of persistent lifelong neurogenesis: the subventricular zone and the dentate gyrus (DG). These regions contain populations of quiescent neural stem cells (NSCs) that generate mature progeny via rapidly-dividing progenitor cells. We hypothesized that the energetic demands of highly proliferative progenitors generates localized oxidative stress that contributes to ROS-mediated damage within the neuropoietic microenvironment. In vivo examination of germinal niches in adult rodents revealed increases in oxidized DNA and lipid markers, particularly in the subgranular zone (SGZ) of the dentate gyrus. To further pinpoint the cell types responsible for oxidative stress, we employed an in vitro cell culture model allowing for the synchronous terminal differentiation of primary hippocampal NSCs. Inducing differentiation in primary NSCs resulted in an immediate increase in total mitochondria number and overall ROS production, suggesting oxidative stress is generated during a transient window of elevated neurogenesis accompanying normal neurogenesis. To confirm these findings in vivo, we identified a set of oxidation-responsive genes, which respond to antioxidant administration and are significantly elevated in genetic- and exercise-induced model of hyperactive hippocampal neurogenesis. While no direct evidence exists coupling neurogenesis-associated stress to CNS disease, our data suggest that oxidative stress is produced as a result of routine adult neurogenesis.

## Introduction

The oxidation state of the normal and diseased postnatal CNS has become the subject of increasing interest in recent years. The brain represents a highly oxidized environment that is particularly vulnerable to oxidative stress due to the brain’s high oxygen consumption rate, its abundant lipid content, and the relative paucity of antioxidant enzymes compared with other tissues [1]. Within the CNS the balance of oxidative stress between the generation and degradation of ROS is tightly controlled [2], and the disruption of this equilibrium has been suggested as a contributor to multiple diseases. Oxidative stress has been implicated in a wide variety of CNS disorders, including Parkinson’s disease [3], Alzheimer’s disease [4], [5], [6], [7], stroke [8] and ALS [9]. From an epidemiological perspective, ongoing oxidative stress offers a compelling explanation for the progressive manifestation of psychiatric and neurodegenerative diseases as oxidative damage accumulates. Oxidative stress is also believed to contribute to dysfunction in otherwise normal tissue as a result of ionizing radiation therapy against brain tumors, particularly in dividing cells [10], [11]. With increasing relevance to a wide range of diseases, identifying sources of oxidative stress (and targeting their suppression or ablation of oxidative species) has been a long-held target for pharmaceutical and therapeutic intervention.

Despite extensive interest in the role of oxidative stress in states of injury and disease, limited attention has been paid to the endogenous production of oxidative stress within the normal CNS. Much of the existing research focuses on excitotoxic events (such as stroke), which ignores other energy-intensive mechanisms such as cell division. While the adult CNS is a predominantly postmitotic, several exceptions exist, including proliferating endothelial cells [12], self-renewing microglia [13], and neural stem cells, which reside in the subventricular zone and hippocampus [14]. NSC function, particularly neurogenesis, represents an increasingly prominent contributor to multiple CNS diseases. New neurons in the hippocampus integrate within the dentate gyrus and contribute to complex functional circuits that are important contributors to learning and memory [15], and increased hippocampal neurogenesis has been implicated in human Alzheimer patients, as well as several mouse models for the disease [16]. Interestingly, neurogenesis within the hippocampus and SVZ occurs through rapidly dividing intermediate progenitors [14], an energy intensive process that may transiently exacerbate localized oxidative stress.

Despite the commonality of memory impairment in multiple diseases associated with ROS production, little attention has been paid to the contributions of ongoing neurogenesis to the oxidative stress load within the hippocampal neurogenic niche. We sought to evaluate the production of oxidative stress biomarkers, specifically the oxidized DNA marker 8-hydroxyguanosine (8OHDG) and oxidized lipid marker 4-hydroxynonenal (HNE), within the hippocampus. While pathological markers for oxidative stress are found throughout the cortex, the highest concentrations of oxidized lipid and DNA were localized within the hippocampus, more specifically, within the SGZ of the dentate gyrus (DG). As this region corresponds to the neuropoietic niche for hippocampal stem/progenitor cells (so-called type I cells, which we refer to hereafter as ‘NSCs’), we hypothesized that the constitutive cell division within the DG was a source of oxidative stress. To address this question, we isolated undifferentiated NSCs from the SGZ and induced them to terminally differentiate. Differentiating NSCs generate intermediate precursor cells that rapidly divide to generate postmitotic progeny in a manner closely matching that described for hippocampal neurogenesis in vivo and in vitro [15], [17]. We assayed differentiating hippocampal NSCs for proliferation rate, differentiation markers and oxidative stress production. During differentiation, intermediate precursor cells – which bridge undifferentiated neural stem/progenitor cells and their postmitotic progeny – exhibited the highest levels of proliferation (as measured by thymidine analog incorporation) and pro-growth mitogen expression. These cells also displayed the highest levels of cellular mitochondria and oxidative stress load compared to more- or less-differentiated counterparts, suggesting that the majority of oxidative stress is generated during a transient window of elevated proliferation accompanying normal neurogenesis.

To further evaluate the relationship between proliferation and oxidative stress within the hippocampus, we putatively identified a set of genes that are positively regulated in response to oxidative stress. We established a set of 20 such genes, and found that the majority of these were statistically upregulated in a mouse model displaying hyperactive levels of basal hippocampal neurogenesis. In addition, animals with exercise-enhanced hippocampal neurogenesis (as described in [18]) exhibited a significant elevation in the majority of genes in this stress-responsive library. When systemically treated with an established antioxidant, the expression of oxidative-responsive genes was markedly lower than vehicle-treated animals, suggesting this gene set is responsive to oxidative stress. Finally, we suppressed neurogenesis in vivo by infusing the antimitotic agent Ara-C into the brains of C57/Bl6 mice. Ara-C-treated mice exhibited dramatically lower levels of neurogenesis and pathological stress markers than vehicle-treated controls, with little change in senescence or gross hippocampal morphology. We conclude that routine postnatal neurogenesis is a significant (albeit normal) source of oxidative stress that may contribute to CNS disease and/or reduced levels of neurogenesis and reduced learning and memory with increasing age.

## Results

**Figure 1:**
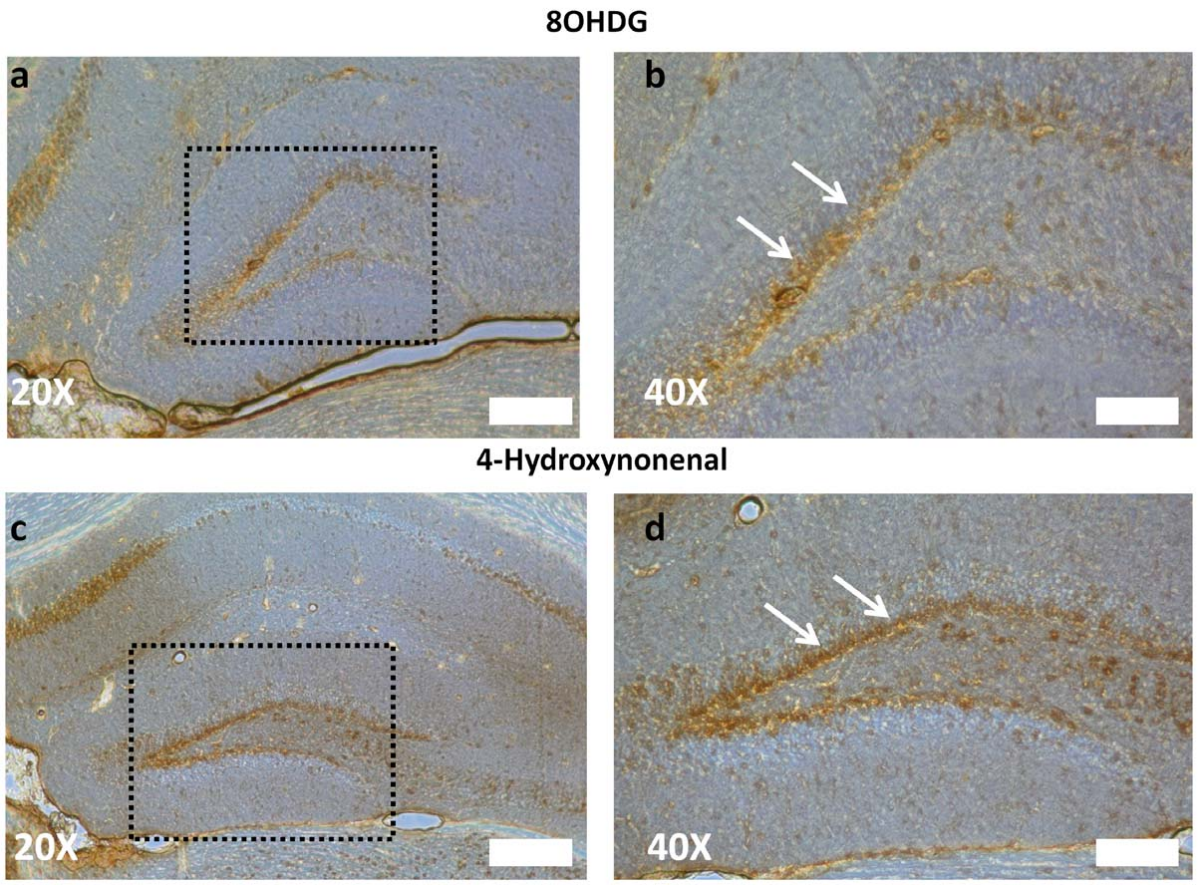
Markers of oxidative stress accumulate in and around the dentate gyrus of the hippocampus. (a) Oxidized 8-deoxyguanosine (8OHDG), a marker of oxidized nucleic acid and (c) hydroxynonenal (HNE), a measure of oxidized lipid, both exhibit localized increases in expression throughout the hippocampus. (b, d) Elevated magnification (boxed areas from a, c) indicated that both these markers express highest levels within the SGZ (arrows). Scale bars: 200 µm (a,c), 100 µm (b,d).

To examine oxidative stress levels in the hippocampus and SVZ, we measured the abundance of oxidized DNA and lipid within neuropoietic zone using antibodies which selectively label the oxidized nucleic acid 8OHDG and oxidized lipid HNE, both established markers for oxidative damage. Both 8OHDG and HNE are highly expressed within many structures of the hippocampus, including the dentate gyrus, CA1 and CA3 (Fig. 1a, c). In particular, the SGZ of the hippocampus – the region in which the primary population of hippocampal neural progenitors reside – exhibited the highest level of oxidative byproducts (Fig. 1b, d, quantification in Fig. S5b). As this region contains actively dividing populations of type I NSCs, we hypothesized that the increased oxidative stress in this structure was associated with these pathological stress markers. We also examined the subventricular niche for similar expression of 8OHDG and HNE. Only moderate labeling was appreciated adjacent to the paired lateral ventricle (data not shown), a disparity that may be due in part to the relatively long distance traveled by rapidly cycling neural progenitors of the SVZ. For this reason, we focused our investigations on the hippocampus.

Next, we sought to examine the association between proliferation and oxidative stress using a two-dimensional model for isolating, expanding and differentiating hippocampal neurogenesis modified from an established protocol [17]. Initial examination of plated NPCs revealed a heterologous population of cells including GFAP(+) astrocytes, nestin(+) immature/progenitor cells, CNPase(+) oligodendrocytes and Tuj1(+) neurons, similar to ratios previously reported from SVZ [19]. A subset of nestin(+) cells expressed the hippocampal progenitor marker Tuc4 (data not shown). Time in culture reduced the percentage of differentiated cells until the majority of cells exhibit immature and/or gliotypic morphologies (Reported in [19] and data not shown).

**Figure 2:**
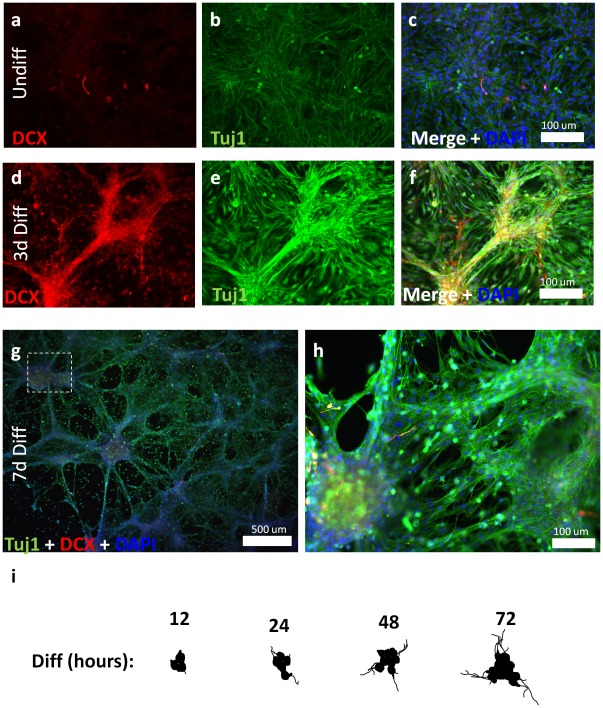
Hippocampal NSCs can be isolated and established as a two-dimensional culture system. (a–c) Undifferentiated hippocampal NPCs are undifferentiated, expressing insignificant levels of Tuj1 and Doublecortin (DCX). (d–f) Three days following initiation of differentiation, individual progenitor cells become Tuj1 (+) and begin to strongly express DCX. (g, h) Neurogenesis proceeds from focal clusters which expand and contact one another within 7 days of induction of terminal differentiation. (i) Camera lucida of representative of expanding clusters of Tuj1(+) neural progenitors at 12, 24, 48 and 72 hours following differentiation.

As reported previously, we found that removal of growth factors (EGF/bFGF, serum) induced a synchronous, terminal differentiation of hippocampal NSCs, resulting in the generation of mature neurons and glia. NSCs mature to predominantly neuronal and astrotypic phenotypes in a time course closely approximating that observed for hippocampal NPCs in vivo [17]. Prior to differentiation, Tuj1 and the early neuronal marker doublecortin are rarely expressed (Fig. 2a–c). Three days following differentiation, cells co-expressing doublecortin and Tuj1 are appreciated (Fig. 2d–f). Doublecortin reaches a maximal expression three days following induction of differentiation, while Tuj1(+) cells expand in number 3–7 days following their induction in a large percentage of cells exhibiting traditional neuronal morphologies (Fig. 2g, h). In passage 3–5 cultures, this differentiation methodology yielded a final population of >50% neurons (as measured by Tuj1 expression; data not shown). NeuN(+) neuronal cells begin to appear seven days following initiation of differentiation (Fig. S1h), and reach a maximal number 20 days after initial differentiation (Fig. S1k), most often in cells displaying traditional granule cell morphology (Fig. S1l). Interestingly, we noted that new neurons occur in clusters of increasing size (Fig. 2i), suggesting a clonal origin to each cluster of neuroblasts where one/few NSCs generate intermediate progenitors that rapidly divide to form clusters of postmitotic neurons. Neurons within these formations extend processes to contact adjacent clusters (Fig. 2g, h). The rates of maturation for new progeny closely match those described for organotypic neurogenesis within the hippocampus [20].

**Figure 3:**
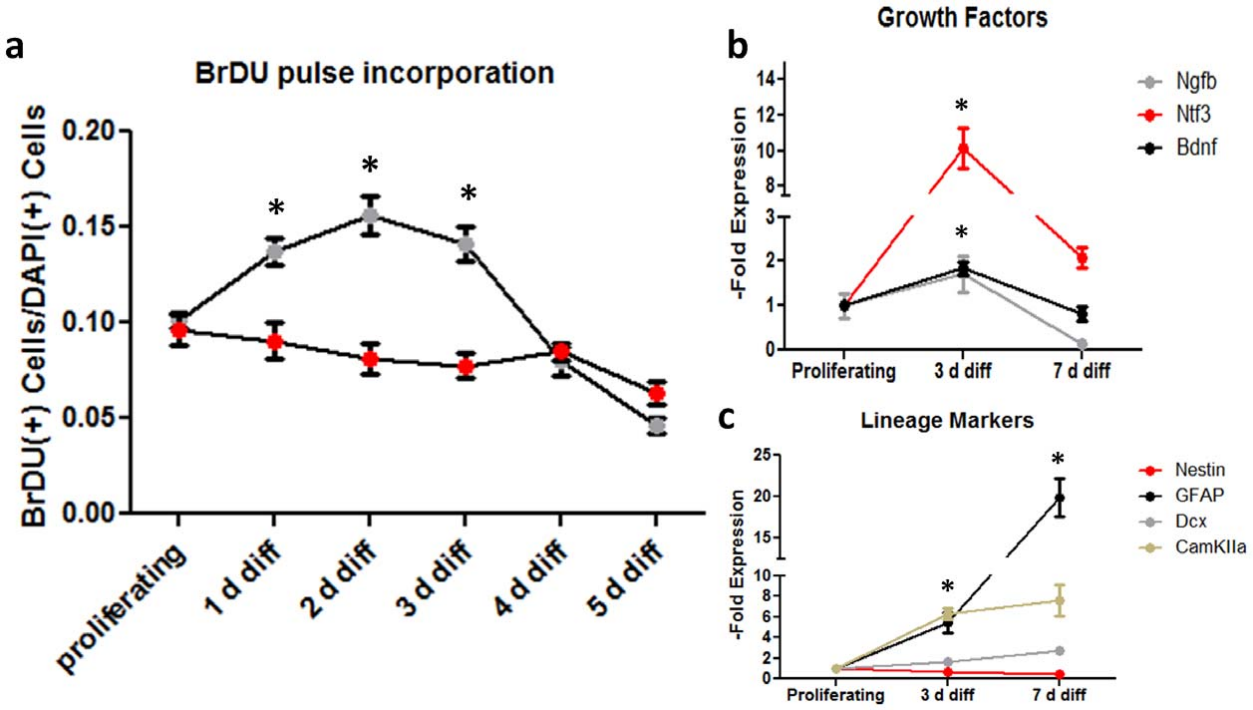
Differentiation of hippocampal NSCs is marked by rapid, transient proliferation and a spike in mitogens. (a) BrdU incorporation was measured by non-overlapping 24-hr consecutive pulse labeling of differentiating and undifferentiated HPCs (see methods). Upon induction of differentiation, hippocampal NPCs (grey data points) significantly increase their proliferative rate for a period of 72 hours when compared to hippocampal NSCs maintained in medium containing EGF and bFGF (red data points). (b) RT-PCR on NPCs revealed a spike (p*<0.05) in expression of several mitogenic factors, including nerve growth factor β (Ngfb), brain-derived neurotrophic factor (Bdnf) and neurotrophin-3 (Ntf33) three days after differentiation was initiated. (c) Terminal differentiation of hippocampal NPCs is accompanied by decrease in the progenitor marker nestin and increases in the lineage markers GFAP and the early- and forebrain- specific neuronal markers Dcx and calmodulin-dependent kinase alpha (CaMKIIα).

To determine the rates of cell division during hippocampal neurogenesis, we examined proliferation rates in differentiating NPCs using non-overlapping BrdU pulse labeling in 24-hour periods. Upon initiation of differentiation, BrdU incorporation significantly increases for 72 hours, reaching a maximum peak 48 hours following initiation of differentiation (Fig. 3a). RT-PCR of mitogenic markers revealed sharp, significant increases in brain-derived neurotrophic factor (Bdnf), neurotrophin-3 (Ntf3) and nerve growth factor (Ngf) in NPCs differentiated for three days compared to more- or less-differentiated timepoints (Fig. 3b). We also confirmed terminal differentiation of NPCs: RT-PCR of differentiating NPCs demonstrates increasing levels of GFAP (glia), Dcx and CaMKIIα (neuronal) with progressive differentiation, while the NSC marker Nestin decreased as differentiation occurs (Fig. 3c). Moreover, the emergence of mature cell types corresponds to a dramatic decline in cell division (Fig. 3a, c). These results indicate that highly active intermediate progenitors generate postmitotic neurons and glia following a transient window of highly mitotic activity 2–4 days after initiation of differentiation.

**Figure 4:**
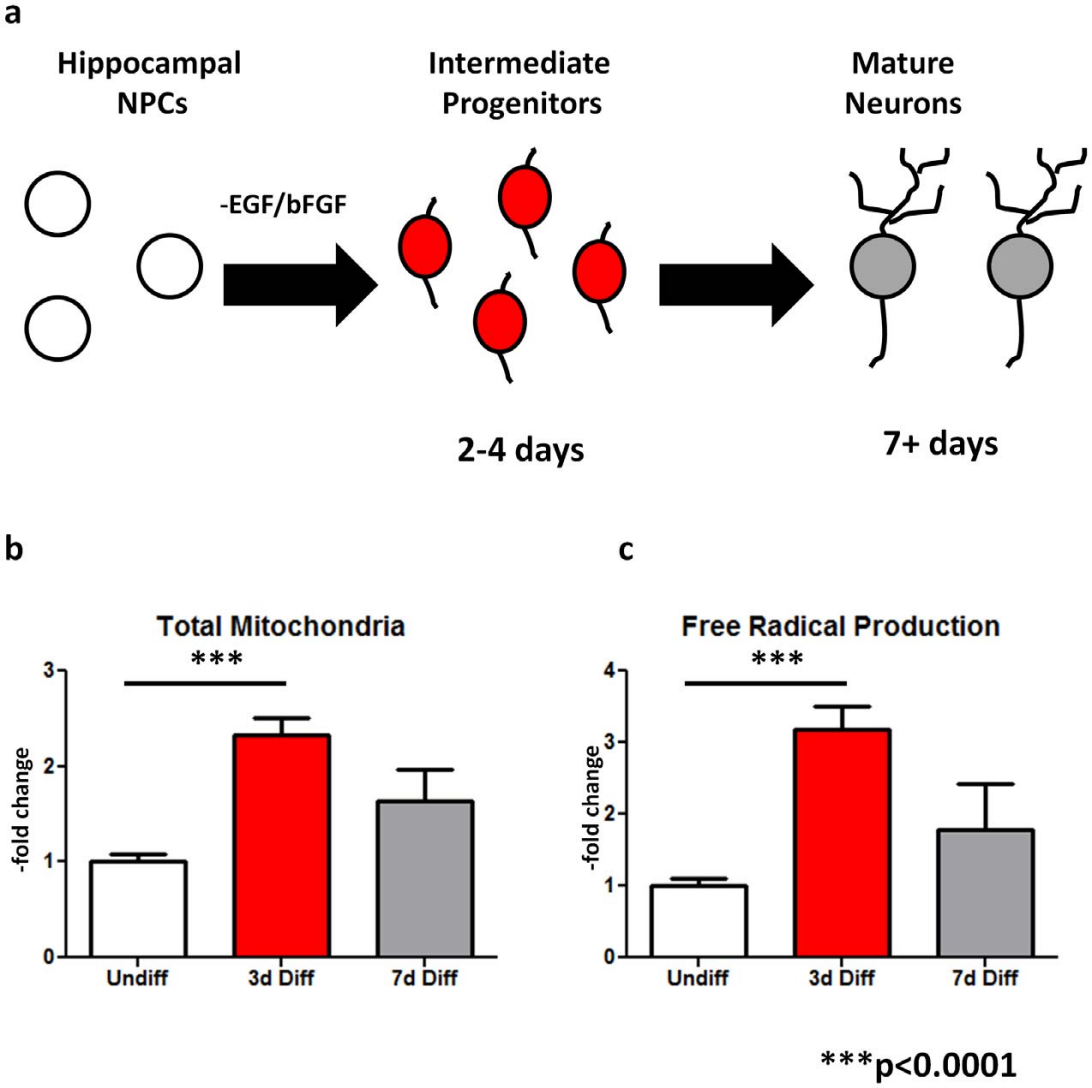
Hippocampal neurogenesis transiently generates oxidative stress. (a) Total mitochondria and oxidative load were measured in undifferentiated NPCs, early progenitors and postmitotic neurons. (b, c) Total mitochondria prevalence was measured via Mitotracker biological dye (b) while total oxidative load was measured using Mitosox biological indicator dye (measured as–fold change compared to undifferentiated NSCs). Quantifiable fluorescence emission measurements demonstrate a significant increase in maximal mitochondrial abundance and peak oxidative load occurs during the period enriched for early progenitors. p*<0.0001.

Next, we sought to correlate heightened levels of proliferation with increased ROS production. Proliferation-associated oxidative stress can be generated by either increased mitochondrial stress or an increase in total number of mitochondria per cell. For this reason, we measured total mitochondrial number and oxidative stress load in differentiating hippocampal NPCs using quantitative indicator dye labeling (see Materials and Methods). Based on our previous findings, we examined three developmental states, including undifferentiated NSCs, highly proliferative transient progenitors (3d post-differentiation) and postmitotic mature cell types (7d post-differentiation; Fig. 4a). Total mitochondrial number was significantly increased during periods enriched for intermediate progenitors (Fig. 4b), as was total oxidative stress load (as measured by ROS production; Fig. 4c). Due to the high initial plating density, total cell number did not significantly change following differentiation of hippocampal NPCs (Fig. S2b), indicating that these data are significant when viewed as absolutes (Fig. 4b, c) or as a ratio of mitochondrial- or oxidative load per cell (Fig. S2c, d). Removal of growth factors from non-neurogenic cortical astrocytes resulted in no significant change in total mitochondrial load or oxidative stress production (Fig. S2e, f). To further confirm the localization of oxidative stress, we rapidly fixed differentiating NPCs labeled with mitochondria and oxidation-sensitive dye, labeled them with markers for HNE and 8OHDG and visualized their co-expression. Mitochondrial and oxidative labeling was greatest within the characteristic “neurogenesis clusters” in differentiating NSCs (Fig. S3a–c), with the expression of HNE and 8OHDG defining individual clusters (Fig. S3b, d). Visible differences in the intensity and pattern of expression distinguish undifferentiated and intermediate progenitor-enriched NPCs (Fig. S3d, e).

To identify which cells in the neurogenesis developmental spectrum generate oxidative stress, we examined co-expression of differentiation-specific markers for NSCs, immature progenitors and mature neurons (corresponding markers: Nestin, PSA-NCAM and Tuj1, respectively) with p22-phox, a subunit of NADPH oxidase whose expression is upregulation is associated with oxidative stress in a broad variety of biological conditions [21]. p22-phox expression was sporadic in undifferentiated NSCs (Fig. S4a–c) and was generally limited to dividing cells (Fig. S4c). Three days after NSCs are induced to differentiate, p22-phox is strongly expressed in clusters of dividing progenitors/neuroblasts (Fig. S4d–f). However, p22-phox is rarely expressed in mature, NSC-derived neurons (Fig. S4g–i). We also examined glutathione oxidation status in differentiating cells using a GSH/GSSG assay that measured the oxidized fraction of the antioxidant peptide glutathione. We found that oxidized glutathione is significantly elevated during neurogenesis (3 days after terminal differentiation of hippocampal NPCs) compared to undifferentiated NPCs (data not shown).

**Figure 5:**
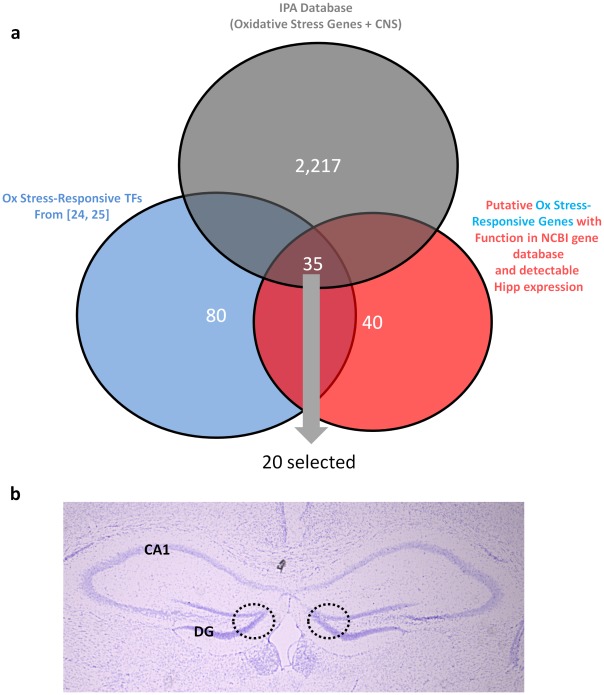
Identification of a set of oxidative stress-responsive (OR) transcription factors. (a) A large subset of genes was identified using the IPA database for genes associated with CNS expression and oxidative stress. This list was compared to two publications ([22], [23]) aimed at large-scale identification of genes which exhibit transcriptional response to oxidative stress. Genes appearing in both data sets were cross-referenced to the NCBI Gene Function Database and the Allen Brain Atlas for acknowledged gene function and detectable hippocampal expression, respectively. Genes appearing on this list were selected *a priori* as potential proxy measures of ambient oxidative stress levels. Gene specific information appears in Table S2. (b) Dentate gyrus was isolated from 500 µm-thick coronal sections using a P300 pipette tip applied to regions shown (dotted line). Sections counterstained with Cresyl Violet.

Technical challenges make direct, real-time measurements (i.e., using biological indicator dyes) within the CNS difficult. To more clearly establish the biological relevance of our findings, we attempted to measure the production of oxidative stress by monitoring a subset of genes that are responsive to oxidative stress within the CNS. To identify a subset of putative OR genes, we searched the Ingenuity Pathway Analysis (IPA) database for genes associated with oxidative stress and expression within the CNS. The resulting list was >2,000 genes (Fig. 5a). We compared this list to gene lists from two notable papers [22], [23] that employed large-scale analysis to identify genes whose transcription is coupled to oxidative stress challenge. Approximately 80 genes were shared by both databases. To further narrow this dataset, we limited this group to genes with established hippocampal expression and discrete functional role (see Materials and Methods). We were able to identify approximately 30 potential oxidation-responsive genes in this manner, of which 20 were randomly selected for study. Oxidation-responsive genes (ORGs) were selected prior to subsequent experiments, with the prediction that they would up-regulate in response to proliferation-associated oxidative stress and down-regulate following removal of this stress.

**Figure 6:**
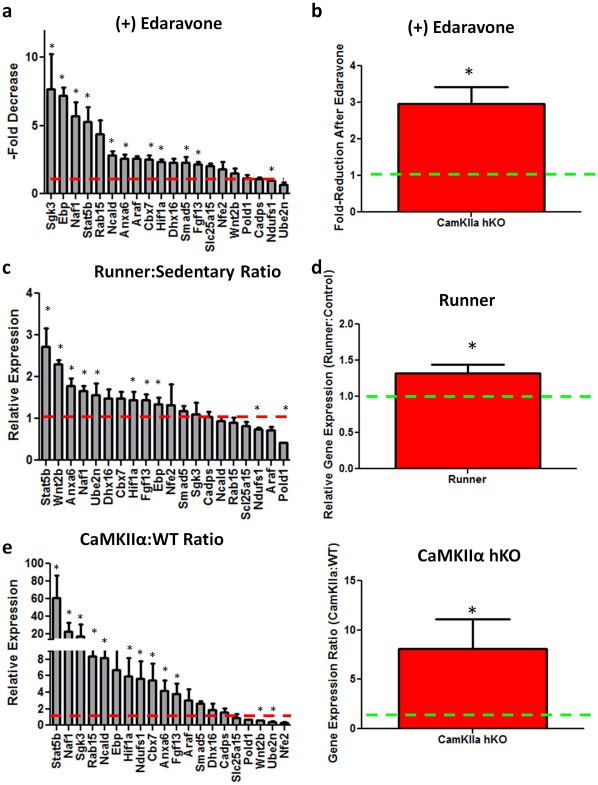
ORGs exhibit elevated levels of expression in multiple models of enhanced neurogenesis, and respond more dramatically to antioxidant administration than normal controls. Infusion of the antioxidant Edaravone (3 mg/kg/day) produces an larger magnitude of decrease in OR genes in CaMKIIα hyper-neurogenic mice compared to controls for both individual genes (16/20; a) and as a group (b). Running (14d) significantly increases the expression of 11/20 ORG members (c), with a significant group increase (d). (e) RT-PCR measures of OR expression. p*<0.05.

To gather data on the stress-associated transcriptional alterations in the dentate gyrus, mice were cannulated and treated with antioxidant or vehicle via osmotic minipump for one week, followed by decapitation. We coronally sectioned the hippocampus and isolated the dentate gyrus using a punch dissection method (Fig. 5b) to examine this region for alterations in ORG expression. First, we attempted to detect the downregulation of ORGs in response to a reduction in oxidative stress, in this case, the systemic administration of the antioxidant Edaravone for one week via subcutaneous minipump. Following antioxidant administration, the majority of ORGs exhibited significantly larger decreases in expression in antioxidant-treated mice than their vehicle-treated counterparts (Fig. 6a). As a group, antioxidant treatment produced significantly larger reductions of expression within the ORG group (Fig. 6b). This finding suggests that this gene set is responsive to reductions in oxidative stress.

Multiple mechanisms have been shown to chemically or genetically enhance neurogenesis, possibly leading to additional oxidative stress. To test the response of ORGs to increased neurogenesis, we elicited increased levels of neurogenesis using unrestricted running, which has been reported to significantly enhance hipocampal neurogenesis [24]. We allowed a cohort of C57/Bl6 mice unrestricted access to running wheels. Mice ran an average of 11.18±2.74 km/day for 14 days, then were immediately sacrificed to measure alterations in ORG expression. Multiple individual ORGs were significantly upregulated in the DG of running mice when compared to sedentary controls (Fig. 6c). As a group, OR genes were significantly elevated in exercising animals (Fig. 6d).

Additionally, we examined ORGs in the DG of a mutant mouse strain reported to possess constitutively elevated levels of hippocampal neurogenesis (the CaMKIIα hKO mouse, generated in [25] and described in [26]). The dentate gyrus of the CaMKIIα hKO mouse is reported to be hyperneurogenic [26]and, as such, represents an area putatively enriched for oxidative stress. Using RT-PCR, we identified multiple ORGs that were significantly upregulated within the dentate gyrus of CaMKIIα hKO mice when compared to WT littermates (Fig. 6e). Group comparison of CaMKIIα hKO and WT littermates revealed a highly significant (>7-fold) increase in ORG family members (Fig. 6f). This suggests an ongoing, localized biological upregulation of oxidative stress-responsive pathways occurs in mice possessing elevated hippocampal neurogenesis. Collectively, these data suggest that (1) ORGs accurately track gene-level responses to positive and negative alterations in oxidative stress, and (2) ORGs are upregulated in response to increased neurogenesis and downregulated in response to antioxidant-mediated suppression of ROS, and (3) Genetic- or behavioral elevation of neurogenesis elicits increases in ORG expression within the DG. We also appreciated individual ORGs frequently respond similarly to different stress-eliciting or –attenuating situations (Table S3).

**Figure 7:**
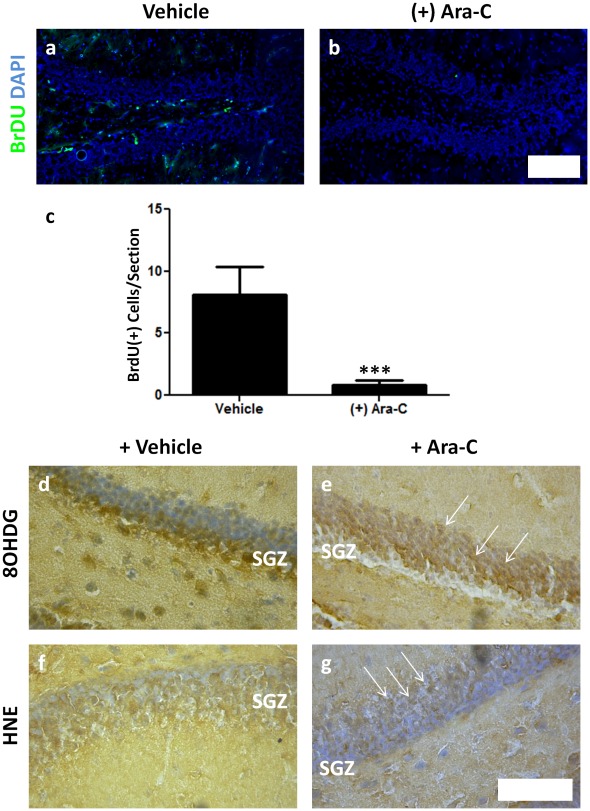
Inhibition of neurogenesis results in the rapid decline of pathophysiological markers of oxidative stress within the SGZ. The antimitotic agent Ara-C or saline (vehicle) was infused twice daily for 7 days prior to thymidine analog labeling, resulting in a significant reduction in total cell division as measured by thymidine analog incorporation (a–c). Acute Ara-C treatment disrupts the production of oxidized DNA (8OHDG; d, e) and lipid (HNE; f, g) within the SGZ. Note existing 8OHDG and HNE (+) cells are retained in the outer granular layer (arrows; DG defined in d–g with Mayer’s hematoxylin). Scale bars: 50 µm (a, b), 100 µm (d–g). ***p<0.001.

To demonstrate the specific relationship between proliferation and oxidative stress, we ablated neurogenesis through direct infusion of the antimitotic agent Ara-C directly into the paired lateral ventricle. We followed a protocol that has been shown effective in ablating neurogenesis within the brain [27], [28]. Following regular infusion of Ara-C for one week, we injected animals with BrdU to assess their levels of proliferation and sacrificed animals 24 hours later. We assessed neuropoietic regions for BrDU incorporation, as well as the histological indicators of histological damage 8OHDG and HNE. Proliferation was dramatically impacted in both the hippocampus (Fig. 7a–c) and subventricular zone (data not shown) following Ara-C infusion. HNE and 8OHDG expression within the hippocampus were both significantly lower in antimitotic-treated animals than in vehicle-treated controls (Fig. 7d–g, Fig. S5b). Specifically, the proliferating SGZ showed a marked decrease in 8OHDG and HNE expression (Fig. 7e, g). We also examined the hippocampus for senescence-associated β galactosidase (SA- β-Gal), a marker for irreversible cellular senescence [29], to evaluate whether cessation of proliferation was accompanied by quiescence. We found no increase of SA- β-Gal cells (data not shown), suggesting that the effect of Ara-C was primarily a result of immediate cessation of cell division and not senescence/quiescence. Taken together, these findings strongly indicate that normal neurogenesis is associated with the generation of oxidative stress, most likely through the developmentally intermediate cells’ proliferation.

## Discussion

We have described the production of ROS as a part of normal neurogenesis. We initially characterized an increase in pathological markers of the oxidative byproducts 8OHDG and HNE. Nucleic acid and lipid oxidation are hallmark to the oxidative damage [3], [30], [31], and our findings agree with clinical findings of oxidative damage in many neurodegenerative diseases. For example, oxidative damage to RNA (but not DNA) has been reported in human hippocampus of patients suffering from psychological disorders [32]. Elevated 8OHDG levels are elevated in individuals afflicted with Parkinson’s disease [3], [30], [33], [34], mild cognitive impairment and Alzheimer’s disease [6], [30]. Other oxidative stress-inducing factors, including hypoxia, have also been shown to increase neurogenesis [35]. In normal rodent brain, we found diffuse, albeit widespread expression of 8OHDG and HNE throughout the neocortex (data not shown). This suggests that oxidative stress is present throughout the CNS, and is not exclusively linked to neurogenesis. Within the DG, we appreciated a mosaic pattern of oxidative stress byproducts in the DG, suggesting the deposition and repair of oxidative damage may be more dynamic than previously imagined. However, the consistently strong labeling the SGZ, indicates that normal neurogenesis is a significant source of oxidative stress. NSCs are unique in that they generate progeny via rapid cell division. For this reason, we hypothesized that heightened levels of proliferation within the SGZ are responsible for the observed increase in 8OHDG and HNE expression.

To investigate the relationship between neurogenesis and oxidative stress, we examined the alterations in proliferation, gene expression, ROS production and total mitochondria in an in vitro model of differentiating hippocampal NSCs that closely mimics in vivo hippocampal NSC behavior. We found that hippocampal neurogenesis peaks 2–4 days after the induction of terminal differentiation. During this period, maximal cell division (as measured by BrDU incorporation) was appreciated, corresponding to a maximal level of total mitochondria and oxidative stress load. Co-localization of the oxidative stress indicator protein p22-phox with neurogenesis developmental markers confirmed that peak levels of stress occur in intermediate progenitors, rather than undifferentiated NSCs or postmitotic neurons. p22-phox expression, mitochondria number, and oxidative stress load decline after this transient period terminates with the emergence of postmitotic progeny. Interestingly, intermediate progenitors are enriched for a number of neurotrophic factors. Considering the well-established relationship between neurotrophins, energy metabolism and oxidative stress within the CNS [36], it appears possible that the rapid proliferation inacted by hyperactive neurogenesis within a relatively quiescent environment may produce excessive localized stress as a consequence.

Under normal conditions, the balance of ROS production and degradation is highly regulated [2]. An imbalance in these mechanisms, either by a lapse in protective mechanisms or a sudden increase in oxidative species production, results in ROS production within the neurogenic niche. As such, high levels of proliferation would place added stress on cells and neuronal circuits within the microenvironment, possibly contributing to the learning and memory functions associated with the hippocampus. Evidence supports this theory: Immature neuronal cell markers, including doublecortin and polysialylated nerve cell adhesion molecule (PSA-NCAM), are increased in the DG of patients diagnosed with Alzheimer’s disease [16]. In spite of these observations, it is important to note that neurogenesis-associated oxidative stress has not been deemed a causal factor of any CNS diseases.

It should be noted that hippocampal neurogenesis is incompletely understood, and that yet-unidentified biological processes may account for a portion of the transient oxidative stress accompanying differentiation. However, by conducting neurogenesis experiments in vitro, we were able to exclude synaptic inputs present in vivo (for example, input from the entorhinal cortex), which reportedly have the capacity to completely deplete the respiratory capacity of individual neurons [37]. This is significant, as ischemia studies have suggested that alterations in mitochondrial energy production under differing conditions (i.e., ischemic vs. reperfused) may lead to the bulk of ROS production [38]. Thus, we were able to rule out synaptic input as a dominant source of oxidative stress while correlating ROS production with elevated proliferation.

Proliferation-associated damage may also contribute to DNA damage leading to immortalizing mutations. Re-entry into the cell cycle is often a precursor step to cell death [39], as well as non-proliferation associated DNA replication leading to aneuploidy [40]. Interestingly, several studies have indicated a unique population of aneuploid cells arising during postnatal neurogenesis [40], [41]. Oxidative stress has been shown to promote aneuploidy [39] and may contribute to these cells in the CNS. Thus, it is possible that ROS contribute to the aneuploidy observed alongside postnatal neurogenesis. ROS-mediated DNA damage is also relevant to tumorigenesis: Glioblastoma multiforme and other grade IV CNS tumors are believed to be the products of malignant NSCs [42]. These tumors appear at an early age, when neurogenesis (and accompanying oxidative stress) is highest. The extent to which oxidative stress contributes to tumorigenesis and aneuploidy in NSCs remains an excellent question for further studies.

To confirm our findings in vivo, we identified a subset of genes that are transcriptionally-responsive to oxidative stress. ORGs expression within the DG was downregulated in antioxidant-treated mice, suggesting they were sensitive to modulation in localized oxidative stress. We noticed several individual ORGs that demonstrated consistent expression patterns in responsive to modulation of neurogenesis. Unsurprisingly, these genes are involved in inflammatory processes throughout the body. For example, Naf1 is highly involved in NF-κB pathway activation in rheumatoid arthritis [43], while Stat5b activation occurs during proliferation/oxidation-associated production of advanced glycation end products [44]. Interestingly, increased neurogenesis, either by unrestricted running or genetic modulation (hyperneurogenic CaMKIIα hKO mice), elicited increases in these genes, suggesting oxidative stress accompanies elevated neurogenesis in vivo. Further evidence of the responsiveness of the ORG family comes in that hyperneurogenic mice do not express elevated levels of HNE and 8OHDG throughout the brain (data not shown), possibly due to overexpression of cellular mechanisms of ROS defense instigated by the observed changes in ORGs. This notion is supported by the fact that many ORGs are involved in proliferative pathways and ROS metabolism (Fig. S2). This defense may come at some cost: Increased ORG expression in running mice is proportionately lower that observed in CaMKIIα hKO mice. Interestingly, runner mice are reported to possess enhanced learning and memory [18], [24], while CaMKIIα hKO mice display deficits in these areas [26]. It is very possible that the cognitive benefits of intermittent exercise on neurogenesis become deleterious when overexpressed in a genetic model.

As a group, ORGs responded positively to gene- and behavior-driven neurogenesis increases, and were attenuated by antioxidant. This suggests that this panel (1) effectively labels alterations in oxidative stress and (2) demonstrates that changes in oxidative stress are, within the hippocampus, in part driven by alterations in neurogenesis. The correlation between oxidative stress and proliferation underscores other relationships previously described between cell cycle and disease. Multiple studies observe cell cycle deregulation as a linking factor between oxidative stress and Alzheimer’s disease [39]. Similarly, oxidative stress has been attributed to lead to cell death and apoptosis [45], both frequently observed (and considered normal) in postnatal neurogenesis, making it unclear how many cells die following exposure to ROS. Cells typically exhibit variegated responses to ROS, including proliferation, growth arrest, or cell death, depending upon which part of the cell cycle is currently underway [46]. The role of oxidative stress in neurogenesis-associated cell death is an interesting question for future study.

Finally, to examine the association between proliferation and ROS production, we inhibited cell division within the DG using the antimitotic agent Ara-C. Compound administration resulted in a dramatic reduction in neurogenesis and reduced the prevalence of HNE and 8OHDG labeling, particularly within the NSC-rich SGZ. Quantification of 8OHDG revealed significant differences in 8OHDG between the inner (SGZ-containing) DG and the outer granule cell layer (Fig. S5b), as well as a significant decrease in 8OHDG expression in the inner DG following 7 days of Ara-C administration (Fig. S5b). In infused animals, we observed some differences in pattern and consistency of oxidative stress byproduct deposition within the SGZ. This may be a result of surgical implantation of cannulae, compound infusion, or side effects from Ara-C and BrdU independent of their action on proliferation. Similarly, the effects of these compounds may have an unexpected consequence of attenuating oxidative stress markers by killing cells in the SGZ. This possibility is remote, however, as Ara-C acts only upon dividing cells, and only a minority of subgranular cells are dividing NSCs/progenitors.

It is important to note that the increases in oxidative stress are transient, and appear to exhibit maximal levels for only a few days. This may be reflective of a biological situation in which transient oxidative stress bear few negative consequences. Some evidence exists indicating that NSCs are well-adapted to handling ROS, such as the finding that grafted NSCs protect their host environment from oxidative stress [47]. As a counterpoint, ROS exposure produces cumulative and long-lasting changes, such as genomic mutations [48]. It is also possible that some synergistic effect exists between ROS damage and downstream function, such as oxidative stress-damaged neuroblasts incorporating into a functional circuit that is already abnormal.

In conclusion, we have described pathological-, molecular- and gene-expression alterations suggesting that oxidative stress is generated during routine proliferation-associated neurogenesis. While wild-type animals exhibit no obvious deleterious short-term effects from this stress, long-term chronic oxidative stress may play a role in late-onset/progressive CNS diseases and loss of function in normal aging.

## Materials and Methods

### Animal Handling

Young adult (P90) C57/Bl6 mice we used for initial characterization of oxidative stress. Calcium/calmodulin-dependent protein kinase II alpha (CaMKIIα) heterozygous knockout mice were generated and described previously [49]. Mice were singly housed following weaning and maintained under standard housing conditions. All protocols involving animals were developed and conducted in accordance with institutional IACUC protocols. Animals were deeply anesthetized with ketamine-xylazine (80- and 5 mg/kg, respectively) and sacrificed prior to tissue collection. For antioxidant administration studies, Edaravone (3 mg/kg; Tocris; Ellisville, MO) or vehicle was administered systemically via osmotic minipump (Alzet; Cupertino, CA) implanted intraperitoneally into anesthetized animals (n = 6 per group). For immunohistochemistry studies, cardiac perfusion was performed using ice-cold 4% paraformaldehyde. Brains were removed and placed in 4% paraformaldehyde overnight, then transferred to 30% sucrose solution. For NSC isolation, sacrificed animals were rapidly decapitated and brains were processed as described.

### NSC Isolation and Propagation

Protocols for isolating, expanding and differentiating progenitor cells from the rodent hippocampus are already well-established. We employed a protocol modified from Scheffler *et al* ([19]) and Babu *et al* ([17]). Briefly, animals (n = 6–8) were anesthetized, sacrificed and rapidly decapitated. Brains were removed and stored in ice-cold PBS. Hippocampal NSCs were microdissected under a surgical microscope. To avoid cross-contamination of biologically distinct stem cell populations (i.e., SVZ- and hippocampal-derived) brains were coronally blocked at the anterior of the hippocampal capsule. To isolate hippocampal NSCs, the posterior region was further coronally sectioned and the hippocampus microdissected from the third ventricle and surrounding cortex. Cortical astrocytes for control experiments were isolated from the pial surface of the distal frontal cortex and processed identically to NSCs.

Tissues were placed in ice-cold Neurobasal Media (Gibco; Carlsbad, CA) containing 3X antibiotics (Gibco). Tissue was manually dissociated with a scalpel into 1 mm^3^ pieces, then placed into anti-adhesive 35-mm culture dishes (Corning; Corning, NY). Tissue was dissociated into a single-cell suspension using a commercially available papain-based neural dissociation kit (Miltenyi; Cologne, Germany). Samples were resuspended in Hank’s Balanced Salt Solution (Invitrogen; Grand Island, New York) and filtered through a 30-µm cell strainer to obtain a final solution.

For establishment and propagation, hippocampal NPCs were seeded at an identical density on plastic dishes coated with poly-D-ornithine and laminin (BD Biosciences; Bedford, MA). Growth medium consisted of Neurobasal medium supplemented with B27 supplements (Invitrogen), 2 mM Glutamax (Invitrogen), 1X penicillin/streptomycin, epidermal growth factor (EGF; 20 ng/ml; R&D Systems; Minneapolis, Minnesota), basic fibroblast growth factor (bFGF; 20 ng/ml; R&D Systems) and bovine pituitary extract (0.5% v/v; Invitrogen). Growth factors were supplemented every other day. Cells were passaged with 0.05% trypsin every 3–4 days. Under these conditions, hippocampal NSCs were expandable for up to 10 population doublings, after which they were not evaluated. For neurogenesis studies, NSCs were plated onto glass coverslips coated with poly-D-ornithine and laminin at a density of 2.5×10^5^ cells/cm^2^ overnight in defined growth medium. To induce differentiation, serum, EGF and bFGF were removed from the culture media. During differentiation, a 50% media change was performed every third day. For in vitro experiments involving thymidine analog labeling, BrdU was prepared in 0.9% saline and applied to culture media (10 uM) as described. To assess changes in proliferative dynamics over time, BrdU was added to cells (n = 3 per experiment) for 24-hour periods, beginning 24 hours prior to differentiation. Following each period of administration, cells were immediately fixed for analysis.

### Immunohistochemistry

Serial 10 µm coronal sections (n = 6 animals unless otherwise noted) were cut on a Leica CM 3050 S freezing microtome (Leica Microsystems; Wetzlar, Germany) and stored at 4 C in cryoprotectant (0.1 M phosphate buffer containing: sucrose (30% w/v), ethylene glycol (30% v/v), polyvinyl-pyrrolidone (PVP-40; 1% w/v). For analysis, sections were removed from cryoprotectant, washed twice in phosphate buffered saline (PBS, pH 7.4) and pretreated with PBS containing 0.3% Triton X-100. Tissues were blocked for one hour at room temperature in PBS containing 0.05% Triton X-100, 5% normal donkey serum and 5% normal goat serum. Primary antibodies included: BrDU (rat monoclonal, 1∶500, Abcam; Cambridge, MA), Doublecortin (rabbit polyclonal, 1∶600, Cell Signaling Technologies; Danvers, MA), HNE (rabbit polyclonal, 1∶600, Alpha Diagnostic International; San Antonio, TX), Nestin (mouse monoclonal, 1∶700; Millipore), NeuN (mouse monoclonal, 1∶500, Millipore; Billerica, MA), 8OHDG (goat polyclonal, 1∶600; Millipore), p22-phox (rabbit polyclonal, 1∶500; Bioss; Beijing, China), PSA-NCAM (mouse monoclonal, 1∶400; Millipore) and Tuj1 (β-III-tubulin; Aves Labs; Tigard, OR). Primary antibodies were applied overnight on an orbital shaker at 4 C. Sections were washed three times in PBS, then incubated with secondary antibodies on an orbital shaker for one hour at room temperature. Secondary antibodies included goat anti-rat, Cy2/3-conjugated donkey anti-mouse, Cy2/3-conjugated donkey anti-rabbit (1∶600; Jackson Immunoresearch; West Grove PA), and horseradish peroxidase-conjugated donkey anti-goat and anti-rabbit (both 1∶600; Santa Cruz). Primary and secondary antibodies were diluted in PBS containing 5% normal donkey serum. Fluorescently labeled samples were washed three times in PBS and mounted on glass slides; HRP-labeled sections were washed in PBS, counterstained with Mayer’s hematoxylin, and labeled using the Impact DAB kit (Vector Labs; Burlingame, CA). SA-β-Gal was detected using a modified protocol from that described in Dimri *et al*. Briefly, cells were fixed in PBS containing 2% formaldehyde and 0.2% glutaraldehyde (25°C, 15 minutes). Following a wash in PBS, cells were incubated (37°C, 6 hours) with fresh SA-β-Gal solution [29]. Stained sections were washed, mounted and visualized on a Leica DMIL LED Microscope. For BrdU imaging, cells were pretreated with sodium chloride/sodium citrate (SSC)-formamide (1∶1, 37C, 2 hrs), washed three times for 10 minutes in SSC, incubated in 2 N HCl (37C, 30 min) and washed with 0.1 M borate buffer (25°C, 10 min). Adherent NSC/NPCs were grown as described, seeded onto glass coverslips coated with poly-D-ornithine and laminin, fixed with 4% paraformaldehyde and labeled as described for tissue sections. Labeled samples were mounted in Vectastain anti-fade media containing DAPI (Vector Labs, Burlingame, CA) and visualized on a BZ-9000 microscope (Keyence; Chicago, IL). For unbiased stereology, every third hippocampal section was selected for analysis. BrDU-positive cells were manually counted and expressed as cells/section. Data was analyzed using Graphpad software (La Jolla, CA).

### Oxidative Stress Measurement and Quantitation

Total mitochondrial number was quantitated using MitoTracker selective intercalating dye (M7514; Invitrogen). To estimate overall free radical production, we employed MitoSox biological indicator dye (M36008; Invitrogen). Stock concentrations of both dyes were prepared according to manufacturer’s instructions in DMSO and diluted to working concentrations in Neurobasal media. Prior to each experiment, cells were washed in PBS and growth media was replaced with dye-labeled media. Mitotracker and MitoSox values were read after 30- and 10 minute incubation periods, respectively, under growth conditions (37C, 5% CO_2_). Fluorescent intensity was measured on a Synergy 4 plate reader (Biotek; Winooski, VT). Indicator dye selectivity was validated using hydrogen peroxide challenge as a positive control. Biological replicates were read in triplicate.

Total and oxidized glutathione was measured using a protocol modified from the GSH/GSSG Glo Assay (Promega). Cells were seeded onto polyornithine-laminin plates and induced to differentiate as described. Oxidized and total glutathione were quantitated in luminescent units using a multiwell plate reader (Biotek) and compared as a percentage of the total oxidized:total glutathione ratio. Data from a minimum of three biological and three technical replicates were used for these experiments.

### Quantitative IHC

Expression of 8OHDG was measured in the DG of C57/Bl6 mice treated with Ara-C or vehicle. DG was separated into inner and outer DG, roughly defined as the inner third (including SGZ) and outer two-thirds, respectively (See Fig. S5). Mice (n = 3 per group) were infused with Ara-C or vehicle as described, sacrificed, and their brains sectioned and assayed for 8OHDG expression using IHC as described. Representative sections of mid-hippocampal coronal sections (n = 3–4/animal) were selected for quantification. Antibody intensity was analyzed using ImageJ and expressed as normalized intensity/area. Individual experimental groups were compared using paired t-test.

### Real-Time PCR

Hippocampal tissue was isolated from the proximal intersection of the dorsal and ventral blades of the dentate gyrus. 500 µm coronal sections were placed under a dissection microscope and tissue punched using a 300 µl pipet. Two punches (four punches total) were isolated from serial sections and homogenized in RLT buffer (Qiagen; Germantown, MD). Cell cultures were washed with PBS and lysed in RLT buffer. RNA was isolated and purified using the RNAeasy Micro Kit (Qiagen) and reverse transcribed using the Superscript III kit (Invitrogen) using random hexamers. cDNA quality/quantity was verified on a Nanodrop 1000 (Thermo Scientific; Waltham, MA).

Real-time quantitative PCR was performed on selected genes using a Viaa 7 analyzer (Applied Biosystems; Carlsbad, CA) using Fast Sybr Green reagent (Invitrogen). Genes of interest were analyzed in duplicate technical replicates, quantified on a standard curve of genomic DNA and normalized to GAPDH or 18 s RNA. Data analysis was performed using Viaa7 RUO software (Applied Biosystems) and statistical significance calculated using Graphpad. Primers for individual genes are listed in Table S1.

### Ara-C Infusion

C57BL/6 mice (n = 3/group) were stereotaxically implanted with permanent unilateral guide cannulae (24 gauge) under a mixture of ketamine (80 mg/kg, i.p.) and xylazine (10 mg/kg, i.p.) anesthesia. Cannulae for the lateral ventricle were inserted according to the following coordinates: 0.4 mm posterior to bregma, 1.1 mm lateral to the midline, and 1.75 mm ventral to the skull surface with no angle. Each cannula was subsequently anchored to the skull by four stainless steel screws and dental acrylic. While the injection cannula (31 gauge), which extended 0.75 mm beyond the tip of the guide, was not in use, a 28 gauge dummy stylet maintained the patency of the guide cannula. Four days after surgery, Ara-C (2% v/v) was infused directly into the ventricle twice daily for 3 days. 48 hours following the initiation of Ara-C treatment, a single BrdU injection (100 mg/kg, i.p.) was administered. Animals were sacrificed 24 hours later and their brains were processed for IHC as described.

### Oxidative Stress-associated Gene Selection

A set of twenty oxidative stress-responsive genes were selected *a priori* using the following criteria: First, the gene expression features of the Ingenuity Systems Pathway Analysis Software (Ingenuity Systems; Redwood City; CA) were mined to generate a list of genes associated with oxidative stress and central nervous system. This gene subset was manually cross-referenced against two large-scale studies aimed at identifying genes upregulated during oxidative stress responses in midbrain dopaminergic [22] and CA1/3 [23] neuronal populations. Approximately 80 genes were present in both groups. A final list of 20 genes was selected by identifying genes that were (a) expressed in the mouse hippocampus at detectable levels (according to the Allen Brain Atlas; http://www.brain-map.org) and (b) linked to one or more discrete functional families in the NCBI gene information database (http://www.ncbi.nlm.nih.gov/gene). Approximately 30 genes meet this criteria, of which 20 were randomly selected for analysis. Individual gene information for oxidative stress-responsive genes are listed in Table S2. Gene expression and significance (Table S3) was calculated singly (against the corresponding control) and as a group analysis (mean of all genes normalized to %±S.E.M. of control) of all genes).

### Running Wheel Analysis

Young adult C57/Bl6 were singly housed for 14 days in standard housing containing wireless running wheels (Med Associates; St. Albans, VT). Running wheel activity was measured in 1 minute binning using proprietary software. Total distance and activity were calculated using Excel.

## Supporting Information

Figure S1 In vitro hippocampal neurogenesis features latitudinal maturation into mature neurons. (a–c) Undifferentiated hippocampal NPCs express only minimal markers of Tuj1 and do not express the mature marker NeuN. (d–f) Expression of mature markers proceeds stepwise: early progenitors express Tuj1 (d) and DCX (Fig. 2d), but not NeuN (e). (g–i) Seven days following differentiation NPC-derived postmitotic neurons begin to express NeuN (h). (j–l) Mature neurons can be identified by NeuN expression (k, 40X magnification of a different field in l), which increases to a maximal frequency approximately 20 days after initiation of differentiation.
(TIF)

Figure S2 (a) Total cell number during differentiation during in vitro differentiation of hippocampal NPCs. NPCs plated at 2.×10^5^ cells/cm^2^ proliferate and are lost at similar frequencies until the generation of postmitotic neurons 5–7 days after the induction of differentiation. At this point, neuronal maturation occurs, with according cell death. (b, c) Total mitochondria number (b) and oxidative load (c) as a ratio of total cell number. Removal of growth factors from similarly isolated non-neurogenic cortical astrocytes did not significantly alter total mitochondria number (e) or oxidative stress production (f).
(TIF)

Figure S3 Mitochondria and oxidative stress are centered within focal clusters of neurogenesis. (a) Mitochondria-specific indicator dye (a, magnified inset in b) and oxidative stress-specific dye (c) demonstrate maximal expression within focal clusters. (d, e) Comparison of oxidation-specific dye signaling intensity in undifferentiated NPCs (d) and cells differentiated three days (e).
(JPG)

Figure S4 The NADPH oxidase subunit p22-phox is transiently increased in differentiating intermediate progenitors. (a–c) Undifferentiated NSCs (identified by nestin expression, b) infrequently express p22-phox (a), which is mainly expressed in dividing cells (arrowheads, c). p22-phox expression is dramatically increased in PSA-NCAM-positive intermediate progenitors (d–f) three days after differentiation. Seven days after differentiation, dividing progenitors generate Tuj1-positive neurons (h), which infrequently express p22-phox (g,i). Scale bar 100 µm (a–i).
(TIF)

Figure S5 8OHDG expression in the DG decreases following Ara-C administration. (a) Dorsal and ventral DG were grouped into inner (inner third of DG, including SGZ) and outer (remaining granule cell layer) layers (shown by dotted lines). (b) Quantification of 8OHDG revealed a significant decrease in expression in the inner (but not outer) DG following 7 days of Ara-C administration. p*<0.05, paired t-test.
(TIF)

Table S1 RT-PCR Primers
(DOCX)

Table S2 Oxidation-Responsive Genes
(DOCX)

Table S3 Oxidation-Responsive Gene Significance. Significance of alteration of ORGs in investigated conditions. Green shading = p<0.05, yellow shading = p<0.1.
(DOCX)
