# Supplementary figures and images for: Adult Neurogenesis Transiently Generates Oxidative Stress
Source: PLoS One. 2012 Apr 30;7(4):e35264. doi: 10.1371/journal.pone.0035264 (PMC3340368; doi:10.1371/journal.pone.0035264)

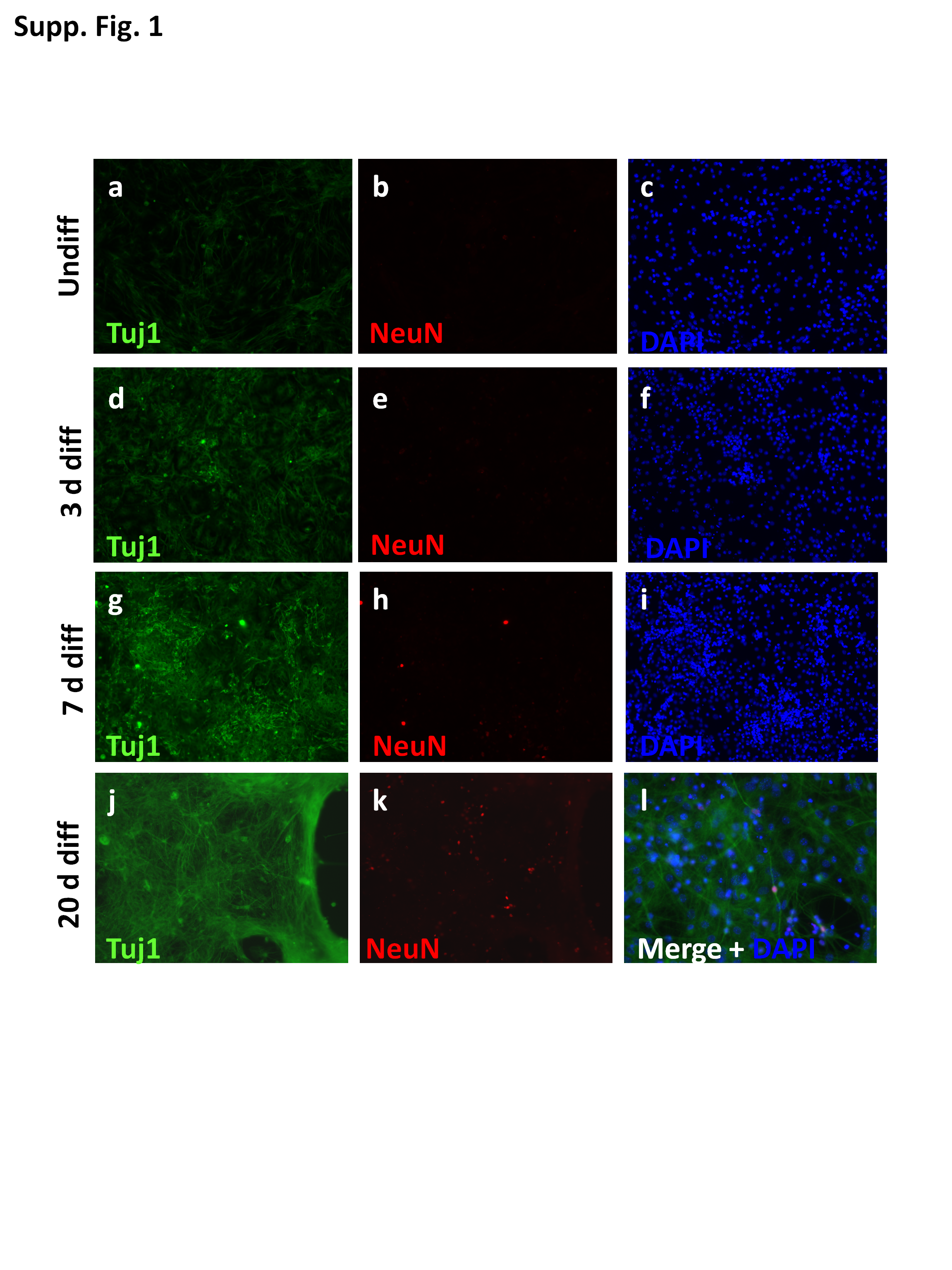

Supplement: Figure S1 — In vitro hippocampal neurogenesis features latitudinal maturation into mature neurons. (a–c) Undifferentiated hippocampal NPCs express only minimal markers of Tuj1 and do not express the mature marker NeuN. (d–f) Expression of mature markers proceeds stepwise: early progenitors express Tuj1 (d) and DCX (Fig. 2d), but not NeuN (e). (g–i) Seven days following differentiation NPC-derived postmitotic neurons begin to express NeuN (h). (j–l) Mature neurons can be identified by NeuN expression (k, 40X magnification of a different field in l), which increases to a maximal frequency approximately 20 days after initiation of differentiation. (TIF) [file pone.0035264.s001.tif]

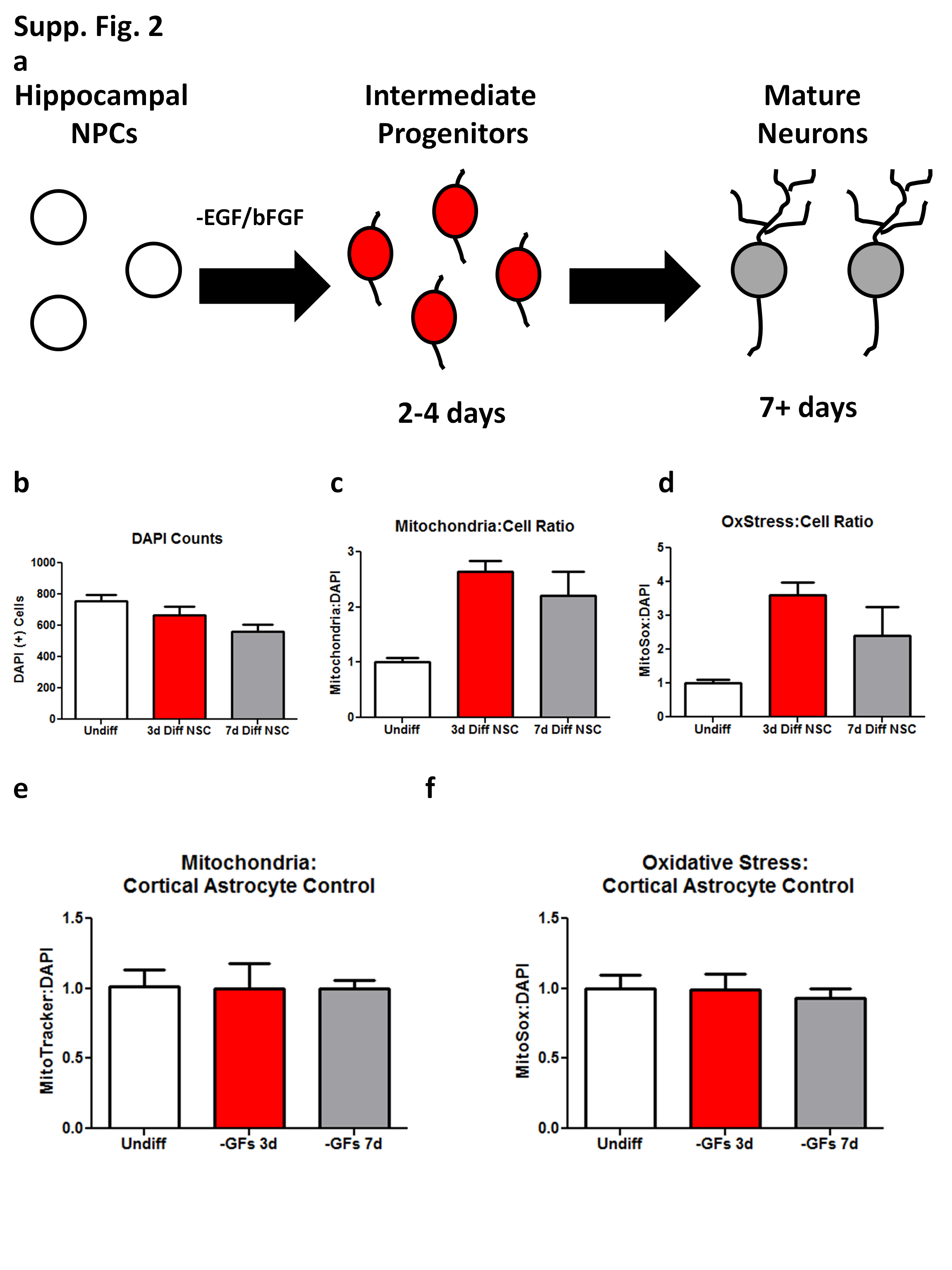

Supplement: Figure S2 — (a) Total cell number during differentiation during in vitro differentiation of hippocampal NPCs. NPCs plated at 2.×105 cells/cm2 proliferate and are lost at similar frequencies until the generation of postmitotic neurons 5–7 days after the induction of differentiation. At this point, neuronal maturation occurs, with according cell death. (b, c) Total mitochondria number (b) and oxidative load (c) as a ratio of total cell number. Removal of growth factors from similarly isolated non-neurogenic cortical astrocytes did not significantly alter total mitochondria number (e) or oxidative stress production (f). (TIF) [file pone.0035264.s002.tif]

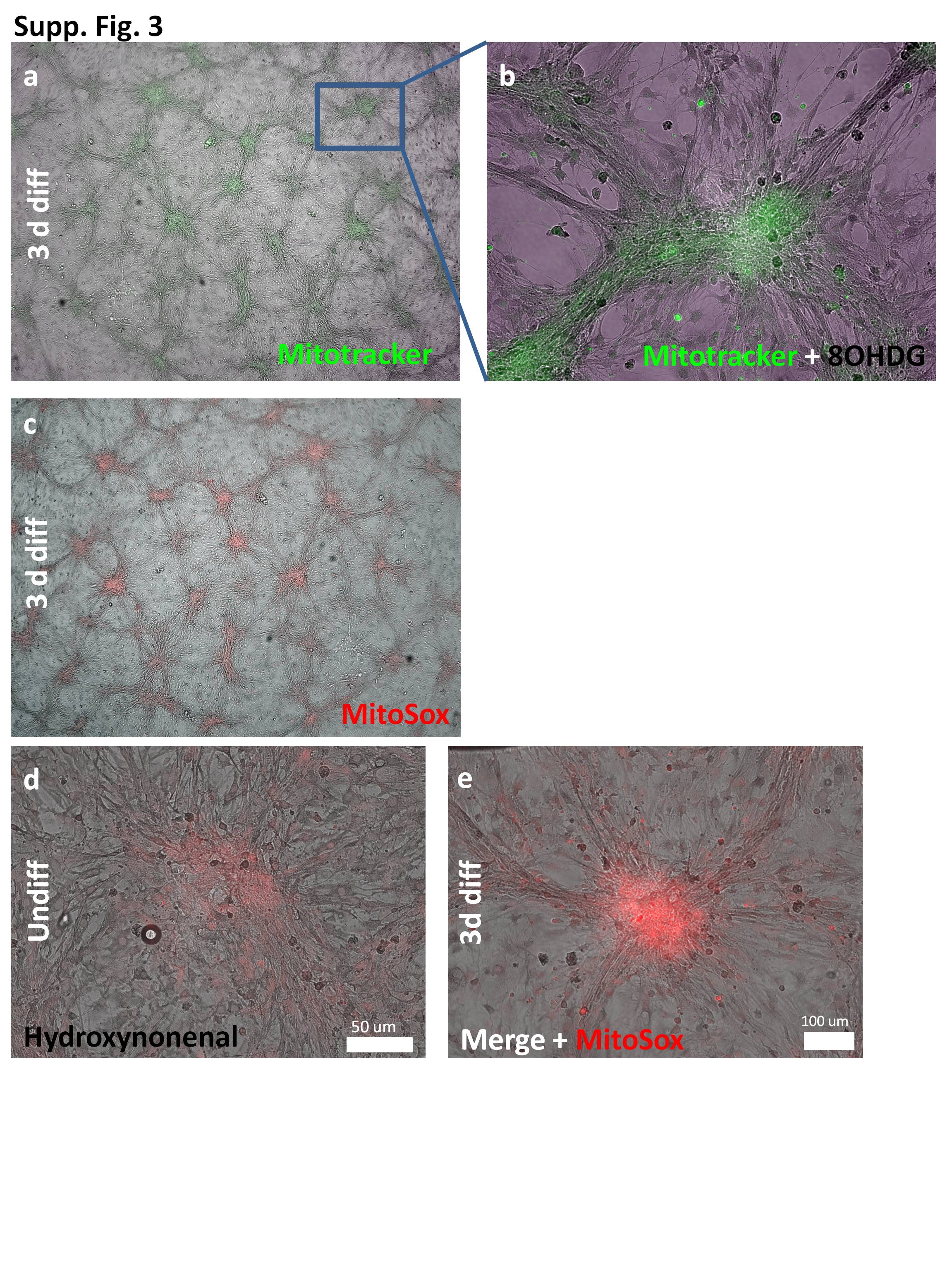

Supplement: Figure S3 — Mitochondria and oxidative stress are centered within focal clusters of neurogenesis. (a) Mitochondria-specific indicator dye (a, magnified inset in b) and oxidative stress-specific dye (c) demonstrate maximal expression within focal clusters. (d, e) Comparison of oxidation-specific dye signaling intensity in undifferentiated NPCs (d) and cells differentiated three days (e). (JPG) [file pone.0035264.s003.jpg]

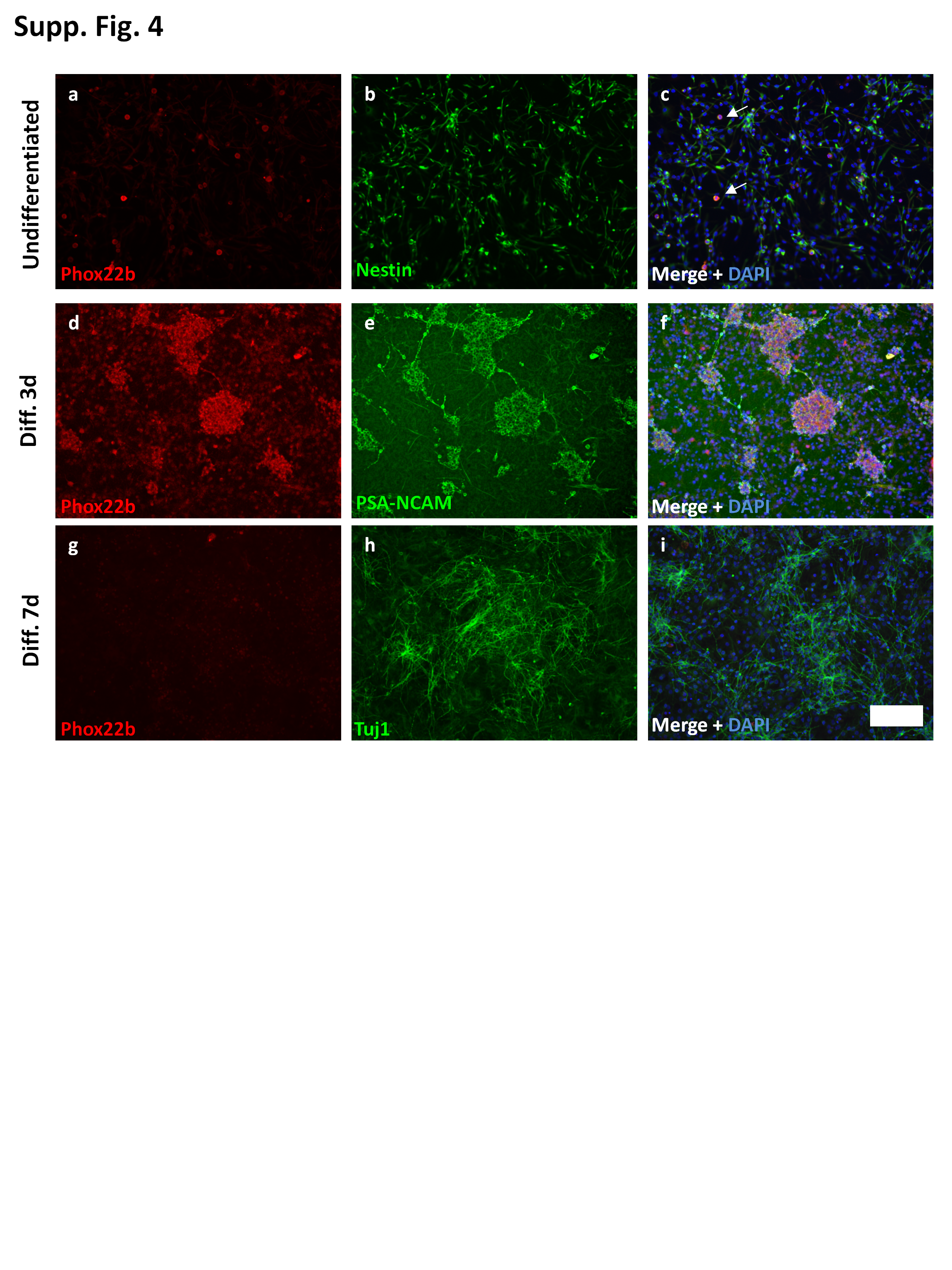

Supplement: Figure S4 — The NADPH oxidase subunit p22-phox is transiently increased in differentiating intermediate progenitors. (a–c) Undifferentiated NSCs (identified by nestin expression, b) infrequently express p22-phox (a), which is mainly expressed in dividing cells (arrowheads, c). p22-phox expression is dramatically increased in PSA-NCAM-positive intermediate progenitors (d–f) three days after differentiation. Seven days after differentiation, dividing progenitors generate Tuj1-positive neurons (h), which infrequently express p22-phox (g,i). Scale bar 100 µm (a–i). (TIF) [file pone.0035264.s004.tif]

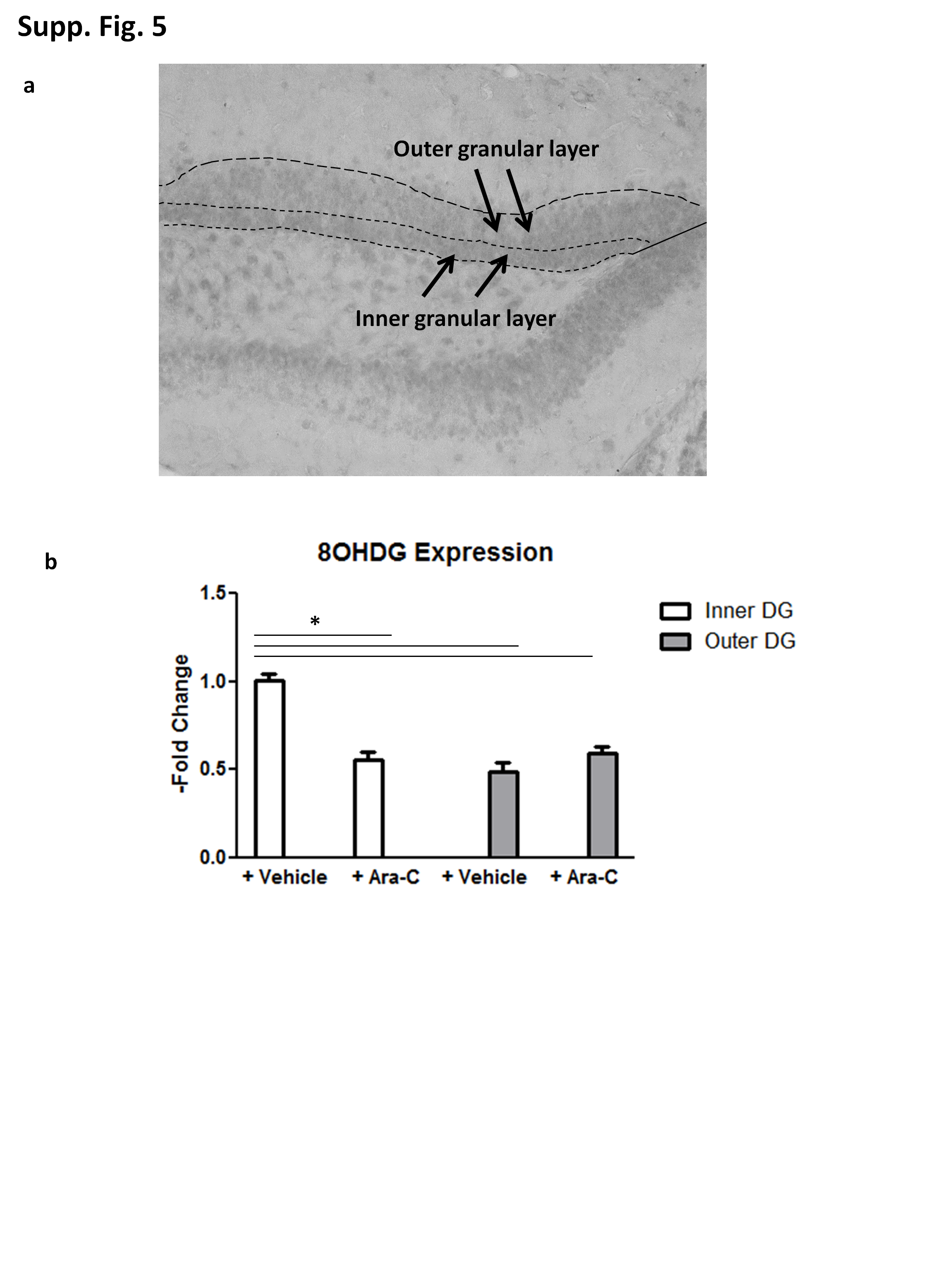

Supplement: Figure S5 — 8OHDG expression in the DG decreases following Ara-C administration. (a) Dorsal and ventral DG were grouped into inner (inner third of DG, including SGZ) and outer (remaining granule cell layer) layers (shown by dotted lines). (b) Quantification of 8OHDG revealed a significant decrease in expression in the inner (but not outer) DG following 7 days of Ara-C administration. p*<0.05, paired t-test. (TIF) [file pone.0035264.s005.tif]
