# Supplementary material for: Adult Neurogenesis Transiently Generates Oxidative Stress
Source: PLoS One. 2012 Apr 30;7(4):e35264. doi: 10.1371/journal.pone.0035264 (PMC3340368; doi:10.1371/journal.pone.0035264)
Supplement: Table S1 — RT-PCR Primers (DOCX) [file pone.0035264.s006.docx]

**Table S1**

| **Gene Name** | **Primer Sequence** |
| --- | --- |
| Hif1a | F-TGCCAGATCACAGCACATTCA  R-TGACACTACAGCAATGCAATGG |
| Cbx7 | \| F-TGGCTGGAGCTTTTCTTTGC \| \| --- \| \| R-GGGATTCTGTCCCCCAATATG \| |
| Smad5 | \| F-CGCCTGTGGGTAGGCAGTAC \| \| --- \| \| R-TCTGTGGTTCAGTGCACACTTG \| |
| Ebp | \| F-GGTGATACCAAGCATCCTTGTG \| \| --- \| \| R-CACGCTCTGGGCACTAGTGA \| |
| Anxa6 | \| F-GTGGCGGAGAGGACTAAAGCT \| \| --- \| \| R-AGTTGGCCACTGCTTGGTAGA \| |
| Nfe2 | \| F-ACAAGGTGGCAGCCCAAA \| \| --- \| \| R-CAGCTGCACAATGGTTTCCA \| |
| Ncald | \| F-GTCCTACAGACGCAGAAAAACAGA \| \| --- \| \| R-TCCCCCACCAAGGATGTG \| |
| Naf1 | \| F-TGTCATGCCCCACTATCCTTTT \| \| --- \| \| R-GCGGAAGTGGAAAGTTATGCATA \| |
| Fgf13 | \| F-CCGGCCGAGGGTGGTA \| \| --- \| \| R-CACATGGTTGCCTTTCATGATC \| |
| Rab15 | \| F-CAGGGAGTCTGGGCTTTGC \| \| --- \| \| R-CTACTGGTTTTCAACAGGATTGTCA \| |
| Cadps | \| F-CCATCCGAAACCGTCTCACT \| \| --- \| \| R-GCCCCCACCCTCACTCA \| |
| Dhx16 | \| F-AAAGTCGGCAAGACACGAGAA \| \| --- \| \| R-AGCTGCAAGGTTTGGTCCAT \| |
| Pold1 | \| F-CCAAGCGCCGCAACTG \| \| --- \| \| R-GCTCCTTGATGGTCGATTACG \| |
| Araf | \| F-CTCCGAACCCTCCTTGCA \| \| --- \| \| R-AAGGCAAGCAGGCAACTCA \| |
| Wnt2b | \| F-CCTCCACTTCAAGCCTCTGACT \| \| --- \| \| R-AGGAAGGTGCGCATGCA \| |
| Sgk3 | \| F-AAAACATATTATGGCTGAACGCAAT \| \| --- \| \| R-CCAACCAAAAATGGGTGCTT \| |
| Ndufs1 | \| F-GCCAAGTGTGTCAAAGCTGTCA \| \| --- \| \| R-AGCATATGGACGGCTCCTCTAC \| |
| Slc25a15 | \| F-GCCCCACGGAGCTTGTG \| \| --- \| \| R-TTTCCTGATGTCTCCATTTCATACA \| |
| Stat5b | \| F-TGGGTTTCGTGAACAAGCAA \| \| --- \| \| R-GTCCCGTCTGGCTTGTTGA \| |
| Ube2n | F-GAGGTGGAGGTTGAACTCTGGTA  R-TCCTGGCCAACGAAAACAG |
| Bdnf | F-GCCCTGCGGAGGCTAAGT  R-GGATGGCCACTCAGAAATTCC |
| Camk2a | F-TGAGAGGAGATTGGAAGGTGTTTC  R-AAGGCATGCGTCCAAGTAGAA |
| Dcx | F-TTCGTAGTTTTGATGCGTTGCT  R-GAGGCAGGTTAATGTTGTCAGACA |
| Gfap | F-TGACCGCTTTGCTAGCTACATC  R-GCGCCTTGTTTTGCTGTTC |
| Nes | F-GGGAGGATGGAGAATGGACTAGA  R-GATCCTCGTCCCCATGCA |
| Ngfb | F-TTCCAGGCCCATGGTACAA  R-GGTGGATGAGCGCTTGCT |
| Ntf3 | F-GGTGGGCGAGACTGAATGA  R-GGGACGTCGACATGAAGAGAA |
