# Supplementary material for: Adult Neurogenesis Transiently Generates Oxidative Stress
Source: PLoS One. 2012 Apr 30;7(4):e35264. doi: 10.1371/journal.pone.0035264 (PMC3340368; doi:10.1371/journal.pone.0035264)
Supplement: Table S2 — Oxidation-Responsive Genes (DOCX) [file pone.0035264.s007.docx]

**Table S2**

| Gene Name | Gene Symbol | Function | Citation(s) |
| --- | --- | --- | --- |
| Annexin VI | Anxa6 | Cell signaling | [23] [50] |
| v-raf murine sarcoma 3611 viral oncogene homolog | Araf | Cell signaling/development | [51] |
| Ca2+-dependent secretion activator | Cadps | Neurotrophin relase/cell survival | [23,52] |
| Chromobox 7 | Cbx7 | Polycomb gene; proliferation/cell cycle | [23,53,54] |
| DEAH (Asp-Glu-Ala-His) box polypeptide 16 | Dhx16 | RNA splicing | [55] |
| Emopamil binding protein | Ebp | Intramolecular Oxidoreductase activity | [23] [56] |
| Fibroblast growth factor 13 | Fgf13 | Mitogen/Cell survival | [57] |
| hypoxia inducible factor 1, alpha subunit | Hif1a | Hypoxia-associated transcription factor | [58] |
| nuclear assembly factor 1 homolog | Naf1 | Robosomal RNA co-factor | [59] |
| Neurocalcin delta | Ncald | Calcium sensor | [23] [60] |
| NADH-ubiquinone oxidoreductase 75 kDa subunit, mitochondrial | Ndufs1 | Oxidoreductase activity, mitochondrial | [61] |
| Nuclear factor, erythroid-derived 2 | Nfe2 | Lineage development/Transcription factor | [62] |
| polymerase (DNA directed), delta 1, catalytic subunit | Pold1 | DNA repair | [23] [63] |
| Ras oncogene family member 15 | Rab15 | RAS oncogene family member | [23] |
| Serum/glucocorticoid-regulated kinase 3 | Sgk3 | Ion channel/transporter regulation | [64] |
| Member mitochondrial carrier; ornithine transporter | Slc25a15 | Mitochondrial transport | [22,65] |
| MAD homolog 5 | Smad5 | Repress cyclin-dependent cell cycling | [23,66] |
| Signal activator and transducer of 5b | Stat5b | Pleiotrophic transcription factor; apoptosis | [22] |
| Ubiquitin-conjugating enzyme E2E 2 | Ube2 | Ubiquitin pathway | [23] |
| wingless related MMTV integration site 2b | Wnt2b | Secreted signaling factor; growth and carcinogenesis | [67,68] |
