# Supplementary material for: Adult Neurogenesis Transiently Generates Oxidative Stress
Source: PLoS One. 2012 Apr 30;7(4):e35264. doi: 10.1371/journal.pone.0035264 (PMC3340368; doi:10.1371/journal.pone.0035264)
Supplement: Table S3 — Oxidation-Responsive Gene Significance. Significance of alteration of ORGs in investigated conditions. Green shading = p<0.05, yellow shading = p<0.1. (DOCX) [file pone.0035264.s008.docx]

**Table S3**

| Gene Symbol | +/- Antioxidant | +/- Running | CaMKIIα hKO/WT |
| --- | --- | --- | --- |
| Anxa6 | 0.01 | 0.004 | 0.02 |
| Araf | 0.08 | 0.16 | 0.14 |
| Cadps | 0.17 | 0.35 | 0.18 |
| Cbx7 | 0.02 | 0.06 | 0.03 |
| Dhx16 | 0.08 | 0.07 | 0.17 |
| Ebp | 0.02 | 0.03 | 0.05 |
| Fgf13 | 0.01 | 0.02 | 0.04 |
| Hif1a | 0.02 | 0.03 | 0.04 |
| Naf1 | 0.03 | 0.001 | 0.04 |
| Ncald | 0.03 | 0.35 | 0.05 |
| Ndufs1 | 0.03 | 0.02 | 0.04 |
| Nfe2 | 0.12 | 0.33 | 0.19 |
| Pold1 | 0.07 | 0.04 | 0.25 |
| Rab15 | 0.06 | 0.33 | 0.05 |
| Sgk3 | 0.05 | 0.38 | 0.05 |
| Slc25a15 | 0.07 | 0.10 | 0.45 |
| Smad5 | 0.04 | 0.22 | 0.09 |
| Stat5b | 0.02 | 0.005 | 0.04 |
| Ube2 | 0.20 | 0.03 | 0.01 |
| Wnt2b | 0.19 | 0.004 | 0.01 |
| Group Analysis | 0.01 | 0.04 | 0.01 |
